# Parental Educational Expectations and Academic Achievement of Left-Behind Children in China: The Mediating Role of Parental Involvement

**DOI:** 10.3390/bs14050371

**Published:** 2024-04-28

**Authors:** Jian Li, Eryong Xue, Huiyuan You

**Affiliations:** 1 Institute of International and Comparative Education, Beijing Normal University, Beijing 100875, China; jianli209@bnu.edu.cn; 2 China Institute of Education Policy, Faculty of Education, Beijing Normal University, Beijing 100875, China; 3 Center for Citizenship and Moral Education, Beijing Normal University, Beijing 100975, China; 202321010016@mail.bnu.edu.cn

**Keywords:** parental education expectation, parental education involvement, academic achievement, left-behind children, migrant workers

## Abstract

Migrant workers from rural China often leave their children at home to be raised by grandparents or other family members. This study explored the relationship between parents’ educational expectations, parental involvement, and the academic performance of left-behind children in China. A total of 19,487 student samples were obtained from the China Education Panel Survey (CEPS), and 5078 of these met the criteria for being considered as ‘left behind’ children. Results indicated: (1) a significant positive correlation between parents’ educational expectations and left-behind children’s academic achievement; (2) parental education involvement plays a partial mediating role between parents’ educational expectations and left-behind children’s academic performance; (3) a significant negative correlation between parental intellectual involvement and educational expectations of left-behind children; (4) parental management involvement was not significantly correlated with parents’ educational expectations and left-behind children’s academic performance; and (5) a significant positive correlation between parental emotional involvement and educational expectations of left-behind children. The findings highlight the important role of parental educational expectations and have implications for the improvement of educational outcomes in China.

## 1. Introduction

Parents play a key role in the growth and development of their children, but for the left-behind children of migrant workers, this input may be lacking [1,2,3]. Left-behind children refer to children under 18 with one or both parents migrating to an urban area for work for at least 6 months [4]. The number of left-behind children in China is very large, with more than 61 million left-behind children in rural areas alone, accounting for almost 22% of the country’s children [5,6]. When parents work away from home, their children are adversely impacted in several ways, including experiencing socio-emotional difficulties, reduced opportunities for family engagement, and exposure to more family conflict. A poor family environment can lead to weak academic performance of left-behind children. The parents of left-behind children leave their young children for a living, go out to work, and earn family income with hard work, but they stay at home in the countryside and spend very little time with their parents, including in mainland cities and some parents go out to work in prosperous cities. These children have become a special group of left-behind children (urban children who do not live with their parents can also be called left-behind children) [7,8,9,10,11].

It was found that there is a relationship between parents’ educational expectations and left-behind children’s academic performance. Higher expectations of family education and active participation of parents are conducive to improving the academic performance of left-behind children [2,12,13]. Migrant work can significantly improve parents’ educational expectations for left-behind children and has a positive impact on their academic performance [14,15,16]. In other words, parents with high expectations for their children tend to be more involved in their children’s education, which enhances their children’s learning engagement and academic performance. Parents’ educational expectations may include academic, career, and life expectations, and children’s abilities can be divided into three dimensions: academic achievement, cognitive ability, and non-cognitive ability. Parental participation has been shown to be a mediating variable in the analysis [17,18,19]. Parental educational expectation has a positive impact on the development of left-behind children’s abilities [20]. When parental involvement is used as a single intermediary variable, parental education expectation increases enthusiasm for parental involvement but has a negative impact on the ability development of left-behind children [21]. In other words, greater parental expectations are linked to more parental involvement but lower academic performance.

**Figure 1:**
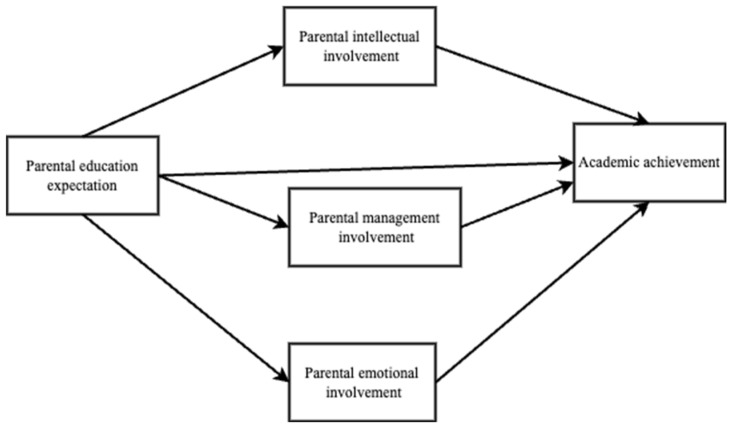
The analytical model of the relations among parents’ educational expectations, parental involvement, and the academic performance of left-behind children in China.

Parental educational involvement refers to several behaviors of parents to enable their children to achieve higher academic results. The phenomenon of parental involvement in children’s education has attracted increasing attention since the 1960s and constitutes an important field of pedagogical research [22,23]. Parental education involvement is of great significance to children’s learning and development. However, few studies have focused on the relationships between parents’ educational expectations, parental involvement, and the academic performance of left-behind children. Therefore, this study aimed to investigate the relationships among parents’ educational expectations, parental involvement, and the academic performance of left-behind children in China, using the analytical model presented in Figure 1 to address the following research questions.

However, most of the current research has been limited to examining the effects of parental educational expectations on children’s academic achievement mediated by parental educational involvement, and most of it has failed to indicate which specific parental educational involvement can improve children’s academic achievement. In addition, there are some contradictory findings, with some studies showing that parental involvement can improve the academic performance of left-behind children, some studies showing that parental involvement is not related to changes in students’ academic performance, and even some studies showing that parental involvement is negatively related to students’ academic performance [24,25]. Therefore, focusing on the points of contention and gaps in current research, this paper explores the impact of parenting expectations on the academic achievement of left-behind children, mediated by specific types of parental educational involvement.

Q1: What is the relationship between parents’ educational expectations and left-behind children’s academic performance?

Q2: What role does parental involvement play in the relationship between parental involvement and left-behind children’s academic achievement? 

Q3: How does parental educational expectation affect left-behind children’s academic achievement?

For Q1, it has been shown that parental educational expectations predict children’s academic achievement, that the association is largely bidirectional, and that this effect varies according to the child’s age, socioeconomic status, ethnicity, type of expectation, and the match between type of expectation and achievement [26,27].

Based on this, we propose:

**Hypothesis 1.**  *Parental educational expectations are positively related to the academic achievement of left-behind children*.

For Q2, parental educational expectations play a direct role in the academic performance of left-behind children via many mediating factors, such as students’ academic self-concept, home-school communication, and parental educational involvement. However, there is considerable inconsistency in the extent and direction of this relationship in terms of the specific role played, with positive, negative, and irrelevant correlations [28]. Regardless of their effects, however, based on these studies, we can formulate:

**Hypothesis 2.**  *Parental educational involvement mediates the relationship between parental educational expectations and the academic achievement of left-behind children*.

As we mentioned above, there is now research that demonstrates that parental educational involvement mediates the relationship between parental educational expectations and children’s academic achievement. However, there is a lack of discussion about the specific effects of this role and the different types of involvement. This is where the greatest innovation of our study lies. Therefore, we propose hypotheses 3–5 in response to Q3. Silinskas, G. et al. (2013) longitudinally compared the academic achievement of first and second-grade children. The results of the three measures indicated that the poorer the children’s reading and math skills, the more homework help and increased supervision their parents would provide, but parental homework help did not positively affect their children’s improvement in related skills and academic achievement [29]. Froiland, J. et al. (2018) studies even indicate that parents’ behavior of checking their children’s homework may have a slight negative impact on their academic performance. Thus, we can see that the mechanism of parental involvement in education on children’s academic performance is complex, and inappropriate involvement in education may have a negative impact on academic performance [30]. Based on the above research, we propose:

**Hypothesis 3.**  *Parental educational expectations negatively affect the academic achievement of left-behind children via the mediating role of parental intellectual involvement*.

Patall, A. et al. (2008) found that parental enforcement of rules about homework (when and where it should be done) had a positive impact on children’s academic performance; home supervision also has a positive impact on children’s academic achievement [31]. Based on this study, we propose:

**Hypothesis 4.**  *Parental expectations are positively related to the academic achievement of left-behind children through the mediating role of parental management involvement*.

Boonk L. et al. (2018) found that parent–child conversations, academic support, and encouragement play a positive role in a child’s academic performance [24]. Based on this finding, we propose:

**Hypothesis 5.**  *Parenting expectations positively affect the academic achievement of left-behind children through the mediating role of parental emotional involvement*.

### 1.1. Parents’ Educational Expectations and Parental Involvement

Parental educational expectation is an important topic in educational literature [32,33,34] because parents’ educational expectations show a significant positive correlation with future educational exploration and investment. Adolescents’ school attitudes can regulate the relationship between the two [35,36]. School attitudes include school liking and school avoidance. Liking school positively predicts exploration and investment in education, whereas school avoidance negatively predicts such outcomes.

Higher parental educational expectations are associated with greater investment in learning by adolescents and their active seeking to achieve educational goals [37,38,39]. The sense of family obligation positively predicts the educational input adolescents receive but cannot significantly predict the number of educational goals attained. Greater parental educational expectations are associated with more in-depth student educational exploration [40,41,42,43]. Academic achievement plays a role in regulating parental educational expectations and future educational outcomes. High academic achievement has a strong predictive effect on parents’ educational expectations for the future educational outcomes of teenagers. Studies suggest that more attention is needed on the influence of parents’ educational expectations, students’ attitudes towards school, family obligations, and other factors to promote the development of future education planning and guidance for adolescents.

Some scholars have focused on the differences in educational expectations between parents and children to promote family harmony, help parents adjust education methods, and ensure children’s healthy growth and academic development. The educational expectations of parents and children can be characterized using four categories, namely, uniformly high educational expectations, uniformly low educational expectations, parents with higher educational expectations (than their children), and children with higher educational expectations (than their parents) [44,45,46].

Parents whose educational expectations exceed that of their children are typically very involved in their children’s academic lives. Teenagers in such families demonstrate good academic performance but poor mental health if they are unable to meet their parents’ high expectations and communicate effectively with their parents to change this situation [47,48,49,50]. Higher performing peers and more frequent interaction between teachers and students mean children’s educational expectations are more likely to catch up with and exceed their parents’ expectations [51,52]. However, too much parental involvement in students’ learning and excessive interaction with teachers and children can have a negative impact on children’s educational expectations. When parents and children have the same high educational expectations, children’s academic performance is optimal and significantly better than in families with a mismatch between parents’ and children’s expectations [53,54,55]. In addition, when parents and children share the same high educational expectations, frequent parent–child communication and companionship can play a positive role in effectively transferring social capital [56,57,58]. Parents and children should communicate clearly to set educational goals because consistent and high educational expectations can help children make optimal progress in learning [59,60].

The relationship between parents’ educational expectations and parent or family social status has also been investigated [61]. Parents with higher education have higher expectations for their children’s education, and these expectations also differ according to occupation [62,63]. Parents’ educational expectations are affected by family socioeconomic level and cultural capital—family socioeconomic status has a negative effect, and the influence of cultural capital is positive—although these effects differ for children of different genders [64,65,66]. Family socioeconomic status has an impact on children’s development, and parents’ educational expectations and personal communication play a mediating role. Parents’ educational expectations have little influence on the development of children whose parents live with them locally and are not migrant workers. However, for migrant children, family socioeconomic status affects development via parents’ educational expectations [67,68,69,70]. The educational expectations of migrant worker parents are often more practical and based on instrumental rationality. They want their children to further themselves and improve their lives. Thus, parents’ educational expectations are also significantly stratified by household registration in China. Parents with different family backgrounds, occupations, and educational qualifications also differ in their educational expectations for their children.

### 1.2. Parents’ Educational Expectations and Children’s Academic Performance

Parents’ educational expectations are an important part of family education [71,72,73]. The relationship between parents’ educational expectations and children’s academic achievement elucidates an understanding of the influence mechanism of family education on children’s academic performance, improving family education methods and helping parents to support their children’s learning and development [74,75,76,77]. Parents tend to put more energy and resources into their children’s learning when they have high educational expectations. This, then, creates a favorable learning environment for their children, which is conducive to the improvement of academic performance [78,79]. However, a threshold effect limits the positive influence of parents’ educational expectations on achievement. That is, when parents’ expectations for their children exceed a certain level, such as hoping that their child can complete a master’s or PhD degree, it is not conducive to the improvement of their children’s academic performance. Parents with high educational expectations for their high school children may increase students’ academic anxiety and have a negative impact on their performance [80]. Parents’ educational expectations can positively predict children’s mathematics achievement, but the motivation-mathematics intermediary chain has a negative effect on mathematics achievement. Differences in the degree of influence of parents’ educational expectations on their children’s mathematical achievement also vary across nationalities. Parents’ educational expectations and students’ self-expectations have a positive effect on academic achievement. Higher parental expectations and academic achievement are associated with students having more positive expectations of going to college.

Academic performance is generally positively correlated with students’ learning engagement. Although some scholars have not directly considered the relationship between parents’ educational expectations and children’s academic performance, they have considered the relationship between parental educational expectations and children’s learning engagement and were, therefore, included in the literature review. Parents’ educational expectations can positively predict student learning engagement, and students’ self-educational expectations play an intermediary role [81].

## 2. Materials and Methods

### 2.1. Research and Analysis Methods

This study used data from the National Survey Research Center (NSRC) at Remin University of China. The China Education Panel Survey (CEPS) was designed and organized by the NSRC. SPSS version 19.0 (IBM Corp., Armonk, NY, USA) was used for data processing, mainly involving descriptive, difference, Pearson correlation, and regression analyses. We also used the PROCESS plug-in of the SPSS macro program compiled by Hayes to construct a model, and the mediation effect was tested using the percentile Bootstrap method of bias correction. Based on the 2013–2014 academic year, the survey investigated junior high school students in Grades 7 and 9 and took the average education level of the population and the proportion of floating population as stratified variables. A total of 19,487 students were randomly selected to participate in the survey. Samples were obtained from 438 classes in 112 schools in 28 county-level units, and the sample was considered nationally representative. For our research purposes, questionnaires that indicated “indifferent” parental educational expectations were removed from the analysis, and a total of 18,645 valid samples were finally included in the analysis model of this study.

### 2.2. Sample Selection

The three types of left-behind children include: those with fathers working away; those with mothers working away, and those with both parents working away. In the CEPS student questionnaire, the question “Which immediate family members are not living with you at home?” is used for screening purposes. Options are coded as follows: “mother” = 1; “father” = 2; “biological siblings” = 3; and “All immediate family members live together” = 4. Students who choose 1 or 2 are defined as left-behind children, with those choosing 1 being the ‘mother away’ type; and those choosing 2, the ‘father away’ type. Students who selected both options 1 and 2 constitute the ‘both parents away’ type. Our research focused on left-behind children, and therefore, we selected samples where students had indicated 1 and/or 2 to obtain a new sample size of 5078.

### 2.3. Measurements

#### 2.3.1. Parental Educational Expectations

Parenting expectations are the highest level of education that parents expect their children to achieve [82]. The item “Your parents’ educational expectations for you” in the CEPS student questionnaire was used to measure parents’ expectations for their children’s future educational level. Response options were encoded: “Now, do not have to read” =1, “junior high school graduation” = 2, “technical secondary school/technical school” = 3, “vocational high school” = 4, “ordinary high school” = 5, “college” = 6, “bachelor’s degree” = 7, “post-graduate” = 8, and “doctoral studies” = 9.

#### 2.3.2. Parental Education Involvement

Parental educational involvement refers to a wide range of behaviors that parents engage in to enable their children to achieve higher levels of academic success [83]. There is no consensus on the concept of parental involvement, and despite its obvious meaning, parental involvement is not clearly defined or operationally defined in a consistent way [27]. To achieve the research objectives of this study, we need to further categorize parental involvement. Most of the existing research is based on the distinction made by Epstein and Comer, which categorizes parental educational involvement into home-based strategies and school-based strategies [24,84,85]. However, this division is a location-based division, not a division based on the type of behavior. Therefore, we borrowed Chinese scholar Song Bing’s division of parental education involvement behaviors and divided parental education involvement into parental intellectual involvement, parental management involvement, and parental emotional involvement and the behavioral scales corresponding to each type of involvement can be found in Table 1 [86]. The Parental Involvement Scale for Junior High School Students (PISJS) has good reliability and validity; each subscale has a high degree of internal consistency, and the scale has good structural and factorial validity and can be used as a valid measurement tool for measuring parental involvement of junior high school students in China. The Parental Involvement Scale for Junior High School Students has good reliability and internal consistency for each subscale and has good structural and factor validity (Table 1).

All the selected sample data used in this study are from the CEPS database, and according to the reasons explained in L495–L500, this study applies the operational definition of the concept of parental participation in education based on Song Bing’s research. The significance of the parental education participation questionnaire compiled by Song Bing is only to help me break down the specific dimensions of parental education participation (parental intellectual participation, parental emotional participation, parental management participation) and the specific behaviors under each dimension (Table 1). Then, based on the survey questions in the CEPS questionnaire, we find similar questions to the parental education participation questionnaire prepared by Song Bing and use the data in CEPS to calculate the parental education participation behaviors of different types of left-behind children. In this study, all my subjects and data used were the same batch of data in CEPS.

For parental intellectual involvement, the corresponding CEPS question is “Did your parents supervise your study in the last week?”, including two items: “Check your homework” and “guide your homework”. The average score of all questions gave the parents’ intellectual involvement behavior score, with higher scores meaning a higher frequency of parental intellectual involvement. The consistency coefficient (Cronbach α) for these questions in this study was 0.76.

For emotional engagement, the corresponding CEPS question is, “Do your parents often discuss the following issues with you? (What’s going on at school, your relationship with your friends, your relationship with your teachers, your mood, and your troubles). The average score of all questions was the parents’ emotional involvement behavior score, with higher scores indicating more parental emotional involvement. Cronbach’s α for these questions was 0.75.

For the dimension of management engagement, the relevant CEPS question was “Are your parents strict about the following things?” (Homework, tests, performance in school, going to school every day, the time you come home every day, who your friends are, how you dress, how much time you spend online, and how much time you spend watching TV). The average score of all questions was the parents’ emotional involvement behavior score, with higher scores indicating greater parental emotional involvement. Cronbach’s α for these questions in this study was 0.86.

#### 2.3.3. Academic Performances

CEPS provides Chinese, math, and English scores for each student, obtained directly from the students’ midterm scores in the fall 2013 semester. The standardized scores of the three subjects are also provided, calculated by school and grade, and adjusted to a mean of 70 and a standard deviation of 10. In this study, academic achievement was defined as the mean of the standardized scores for verbal, mathematical, and English scores provided by CEPS (See Appendix A).

## 3. Results

### 3.1. Difference Analysis of Left-Behind Children

Of the 5078 samples in this study, 52.6% of left-behind children were male, and 47.4% were female. Only children constituted a minority (35.2%). Children with mothers working away were the smallest group (14.0%), and those whose fathers worked away formed the largest group (43.9%), followed closely by those where both parents worked away (42.1%) (Table 2).

Children from different left-behind conditions differed in terms of parents’ educational expectations (F = 23.58, *p* < 0.01), academic standards (F = 17.91, *p* < 0.01), intellectual (F = 104.79, *p* < 0.01), management (F = 13.48, *p* < 0.01), and emotional involvement (F = 33.66, *p*< 0.01), along with significant differences (*p* < 0.01) for other variables. Multiple post hoc comparisons show that. Key findings were as follows: (1) In terms of educational expectations, the scores of non-left-behind children were significantly higher than those of left-behind children; (2) For academic achievement, the scores of left-behind children whose mothers work away were significantly lower than those of other children, and the scores of left-behind children whose fathers work away were significantly lower than those of non-left-behind children; (3) The scores of non-left-behind children for parents’ intellectual, emotional and management involvement were significantly higher than those of left-behind children. For the left-behind children, the scores of those who have both parents working away were significantly lower in terms of parental intellectual involvement than where only one parent was away, and the scores of the left-behind children with mothers working away were significantly lower than the other two categories (fathers away, both parents away) in terms of parental management involvement (Table 3).

### 3.2. Mediating Roles of Parental Intellectual, Management and Emotional Involvement

After controlling for demographic variables such as type, grade, only child, and gender of left-behind children, Pearson correlation analysis was conducted on variables including parental educational expectation, intellectual involvement, emotional involvement, management involvement, and academic achievement (Table 4). We found a significant correlation between parental management involvement and academic standards achievement, as well as academic standards achievement, parental intellectual involvement, parental emotional involvement, and parental educational expectation. A negative correlation was noted between parental intellectual involvement and academic achievement.

We examined the mediating role of parental intellectual involvement, parental management involvement, and parental emotional involvement on parents’ educational expectations and academic achievement of left-behind children using controlling variables such as type, grade, only child, and gender of left-behind children. The regression analysis summarized in Table 4 shows that parental educational expectations positively predicted parental intellectual involvement (β = 0.06, *p* < 0.001), management involvement (β = 0.03, *p* < 0.001), and emotional involvement (β = 0.04, *p* < 0.001). Parental educational expectations can also directly predict the academic performance of left-behind children (β = 0.35, *p* < 0.001). Parental intellectual involvement negatively predicted the academic performance of left-behind children (β= −0.01, *p* < 0.001), and parental emotional involvement positively predicted the academic performance of left-behind children (β = 0.05, *p* < 0.001). However, parental management involvement did not predict the children’s academic achievement significantly (β = −0.01, *p* > 0.05) (Table 5 and Table 6).

**Figure 2:**
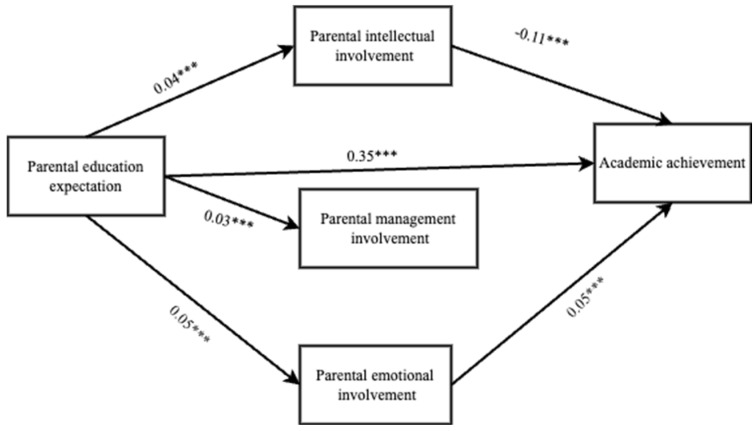
Model of mediating effects of different parental involvement types. *** *p* < 0.001.

Parents’ educational expectations have a significant direct effect on the academic achievement of left-behind children, and parental intellectual and emotional involvement have a partial mediating effect between parents’ educational expectations and the academic achievement of the children. Specifically, the mediating effect is produced by two pathways. Indirect effect 1 is the indirect effect produced by parental educational expectation on parental intellectual involvement and the academic achievement path of left-behind children (−0.04 [95% CI (−0.06–−0.02)]. Indirect effect 3 is produced by parental educational expectation on parental emotional involvement and the academic achievement path of left-behind children (0.04 [95% CI (0.01–−0.06)]. The Bootstrap 95% confidence interval for indirect effects of these two paths did not contain 0, indicating that both indirect effects reached the significance level. However, indirect effect 2, produced by parental education expectation on parental management involvement and left-behind children’s academic achievement path, was −0.01 [95%CI (−0.03–−0.01)] and Bootstrap 95% confidence intervals all contained 0, indicating that this indirect effect did not reach significance (Figure 2).

### 3.3. Influence of Parents’ Involvement on the Academic Achievement of Left-Behind Children

To further compare the influence of parents’ educational involvement on left-behind children’s academic performance, we conducted a correlation analysis of parents’ intellectual, emotional, and management involvement. In terms of management involvement, only three of the eight management measures had a significant positive correlation with the academic performance of left-behind children, namely: parents’ management of homework and exams (γ = 0.067, *p* < 0.001), management of Internet time (γ = 0.051, *p* < 0.001), and management of TV time (γ = 0.029, *p* = 0.044). In addition, there was a significant negative correlation with the academic performance of left-behind children, that is, parental management of returning home time (γ = −0.036, *p* = 0.014), but the correlation was not significant for other items. Two intellectual involvement behaviors, homework instruction (γ = −0.075, *p* < 0.001) and inspection of homework (γ = −0.062, *p* < 0.001), were significantly negatively correlated with the academic performance of left-behind children. For parental emotional involvement, the five emotional involvement behaviors of mothers positively correlated with the academic performance of left-behind children as follows: “Discuss what happened at school” (γ = 0.087, *p* < 0.001), “Talk about your relationship with friends” (γ = 0.036, *p* = 0.19), “Talk about your relationship with the teacher” (γ = 0.064, *p* < 0.001), “Ask about your mood” (γ = 0.046, *p* = 0.003), and “Ask about your heart or troubles” (γ = 0.041, *p* = 0.007). In contrast, only 3 of the 5 emotional involvement behaviors of fathers were positively correlated with the academic performance of left-behind children, namely: “Discuss what happened at school” (γ = 0.037, *p* = 0.016), “Talk about your relationship with the teacher” (γ = 0.035, *p* = 0.023), and “Ask about your mood” (γ = 0.031, *p* = 0.042).

The correlation analysis of specific involvement behaviors for parental management involvement showed 3 measures that were significantly positively correlated with academic performance. The scores of these behaviors were added and averaged, and the new values obtained were assigned to parental management involvement and named ‘Parental management involvement 2’. After these changes, the regression analysis operation was repeated to test the mediating role of parental intellectual involvement, adjusted parental management involvement, and parental emotional involvement with parents’ educational expectations and academic achievement of left-behind children. Parental management involvement 2 positively predicted the academic achievement of left-behind children (β = 0.03, *p* = 0.016). It shows that the adjusted parental management involvement partially mediates the relationship between educational expectation and children’s academic achievement, and the indirect effect of parental management involvement through parental educational expectation, parental management involvement, and left-behind children’s academic achievement path (0.03 [95% CI (0.01–−0.05)].

**Figure 3:**
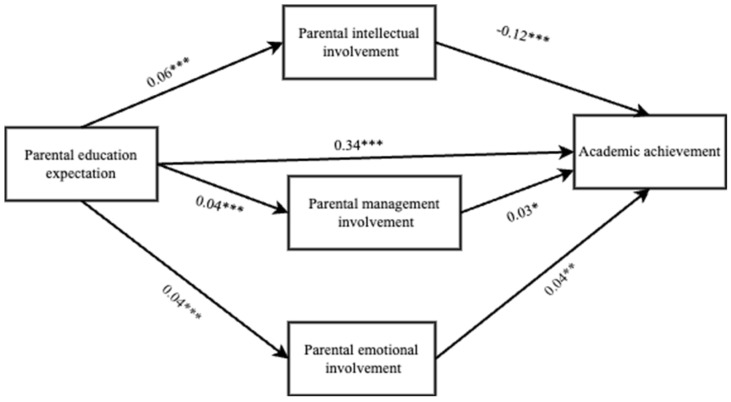
Adjusted mediation effect model. * *p* < 0.05; ** *p* < 0.01; *** *p* < 0.001.

The difference test of indirect effects of different paths showed that the Bootstrap 95% confidence interval [95% CI (−0.02, −0.01)] for the difference between indirect effect 2 and indirect effect 1 did not contain 0, indicating a significant difference between those two indirect effects and that the indirect effect of adjusted parental management involvement was lower than that of parental intellectual involvement (Table 6). The Bootstrap 95% confidence interval [95% CI (−0.02, −0.01)] for the difference between indirect effect 2 and indirect effect 3 did not contain 0, indicating a significant difference between indirect effect 2 and 3, and the indirect effect of adjusted parental management involvement was lower than that of parental emotional involvement. The difference between indirect effect 1 and indirect effect 3 [95% CI (−0.01, −0.01)] did not contain 0, showing that indirect effect 1 was significantly different from indirect effect 3, and the indirect effect of parental intellectual involvement was slightly higher than that of parental emotional involvement (Table 7 and Table 8; Figure 3).

## 4. Discussion

Left-behind children obtained lower scores for parents’ educational expectations, academic standards, and parents’ intellectual, management, and emotional involvement in comparison with children who have not been left behind. These findings are consistent with the results of previous studies that indicate that parents working away from home have a negative impact on the academic development of the children left behind. First, this study found that the family socioeconomic conditions and mothers’ and fathers’ education levels were significantly lower for left-behind children than those of non-left-behind children. According to the theory of cultural reproduction, left-behind children are weaker than non-left-behind children in terms of cultural and economic capital and are academically disadvantaged from the outset. Second, left-behind children are separated from one or both of their parents for a long time, which leads to less communication with their parents and limited emotional support from them [14,15,16].

Parents’ educational expectations have a significant positive impact on the academic performance of left-behind children—a conclusion that is consistent with previous research results [24,82,87]. This also proves that our hypothesis 1 is valid. Parental educational expectations directly impact the academic performance of left-behind children, affecting children via a self-fulfilling prophecy. Higher educational expectations of parents would lead to more involved and supportive parental behaviors to improve the children’s academic performance. Therefore, especially for left-behind children, parents should give their children sufficient educational expectations that are conducive to good academic performance.

Parental educational involvement played a partial mediating role between parents’ educational expectations and left-behind children’s academic achievement. This is also in line with the findings of the study in question [28], Which proves that hypothesis 2 is valid. This shows that parental educational expectations affect children’s academic performance not only by influencing their sense of academic efficacy but also by increasing their engagement behaviors. This also reveals that parents of left-behind children should not only give their children sufficient educational expectations but also externalize this expectation to fully influence their left-behind children via both psychological and behavioral aspects.

Unfortunately, the intellectual engagement behaviors of the parents of left-behind children have a side effect on their children’s academic performance. This finding is in line with the findings of some studies and not so much with the findings of some studies [30,88,89]. Altschul (2011) showed that parents’ help with their children’s homework had a positive effect on their children’s academic performance [90]. This may be due to what Nunez et al. (2015) found in their study that in the act of parents helping their children with their homework if the focus is on controlling the completion of their homework, the role played is negative, while if the supportive dimension of their homework is emphasized, the role played is mostly positive. In addition, this may be due to the level of education of the left-behind children’s parents, most of whom have a relatively lower level of education and are more limited in the methods and content of their intellectual involvement [91]. In addition to this, as other studies have demonstrated, parents provide more homework help and increase supervision when their children’s academic performance is poorer [29]. However, by this time, the child’s grades have dropped. In any case, this also proves that our hypothesis 3 is valid. Therefore, for left-behind children, their parents should control their impulse to become involved in their children’s homework and try to give more supportive encouragement rather than excessive supervision.

Parental management involvement did not play a mediating role, but the other forms of involvement served as mediators. When the definition of parental management involvement was adjusted, parental management involvement also played a partial mediating role. In this study, management involvement was defined using eight behaviors, and no significant relationship between parents’ management involvement and the academic performance of left-behind children was found. Of these eight behaviors, the management of home-time return had a significant negative relationship with academic performance. Parents’ management of homework, exams, internet, and TV time had a significant positive relationship with the academic performance of left-behind children. This suggests that parents who are migrant workers could strengthen the management of their children’s Internet and TV time to cultivate healthy media habits in their children [31,32,33,34]. This partly supports our hypothesis that the effect of parental management involvement on the academic performance of left-behind children is contingent on specific management behaviors.

A significant positive correlation was found between parental emotional involvement and the academic achievement of left-behind children. In the case of long-term separation between children and one or both parents, the emotional intervention of parents has a very important impact on academic performance, including discussions about what happened at school, relationships with friends and teachers, mood, and problems. This finding is consistent with previous studies [92,93,94,95,96,97]. Therefore, our research hypothesis 5 is also proved. This also reveals that parents of left-behind children must pay attention to the emotional involvement in the education of their left-behind children, often talk with them about what happens in school, pay attention to their moods and thoughts, help them make future plans, and solve their emotional-emotional problems in a timely manner.

It is worth noting that during the epidemic period, when left-behind children’s working parents in the cities or provinces were unable to accompany them, millions of rural left-behind children experienced more serious physical and mental distress, with problems such as academic maladjustment and anxiety becoming more serious, and some of the left-behind children even showed serious mental illnesses [98,99]. Due to the impact of the epidemic era, left-behind children need their parents’ emotional involvement more than ever before, and parents of left-behind children must pay attention to satisfying the emotional needs of their left-behind children, which is very important for both their mental health and their academic performance. In addition, due to the online classes in the epidemic era, some left-behind children’s academic situation is worrying, so parents must pay attention to the way of participation in intellectual involvement and focus on the supportive approach.

## 5. Conclusions

The results show a significant positive correlation between parental educational expectations and left-behind children’s academic achievement. Parental educational involvement plays a partial mediating role in the relationship between parental educational expectations and left-behind children’s academic achievement. Parental Intellectual Involvement (checking homework and guiding homework) is significantly negatively related to left-behind children’s academic achievement, mediated by the path from parental educational expectations to parental intellectual involvement to left-behind children’s academic achievement, i.e., the more frequently parents check homework and guiding homework, the more negatively it affects left-behind children’s academic achievement. The correlation between parental management involvement and left-behind children’s academic performance is not significant, specifically for each behavior, including five non-significantly correlated behaviors, three significantly positively correlated behaviors (management of homework and exams, management of time spent on the internet, and management of time spent watching TV), and one significantly negatively correlated behavior (management of time spent at home)There is a significant positive correlation between parental affective involvement and the academic performance of left-behind children, which is mediated by the path from parental educational expectations to parental affective involvement to the academic performance of left-behind children. That is, the higher the frequency of parents discussing the following things with their children, the more positive the effect on left-behind children’s academic performance: what happens at school, your relationship with friends, your relationship with teachers, your mood, what’s on your mind, or what’s bothering you.

Therefore, parents of left-behind children can achieve more obvious educational results by adopting appropriate educational involvement while giving their children educational expectations. Particularly needed is the parents’ emotional involvement; in addition, they can also use part of the management involvement way, such as the management of homework, exams, internet time, and time to watch TV.

## 6. The Practical Implications and Future Recommendations

For practical implications, this study contributes to exploring the relationship between parents’ educational expectations, parental involvement, and the academic performance of left-behind children in China. It is found that there is a significant positive correlation between parents’ educational expectations and left-behind children’s academic achievement. In addition, parental education involvement plays a partial mediating role in the relationship between parents’ educational expectations and left-behind children’s academic performance. There is a significant negative correlation between parental intellectual involvement and educational expectations of left-behind children. Parental management involvement was not significantly correlated with parents’ educational expectations and left-behind children’s academic performance. Additionally, there is a significant positive correlation between parental emotional involvement and educational expectations of left-behind children.

In addition, for future recommendations, we suggest applying more recent and updated data to examine the relationship between parents’ educational expectations, parental involvement, and the academic performance of left-behind children in China. In the meanwhile, more qualitative studies, such as interviews or focus group studies, could be added to investigate how the contextual factors influence the relationship between parents’ educational expectations, parental involvement, and the academic performance of left-behind children in China.

## Figures and Tables

**Table 1:** The Parental Involvement Scale for Junior High School Students (PISJS)by Song Bing.

	Mother	Father
Parental intellectual involvement	Mother studying middle school textbooks or tutorials on her own in order to tutor you Your mother buys books on educating children Your mother attends lectures on education Your mother tutors you in your studies Your mother takes you to libraries, museums, cultural centers, etc. Your mother watches educational TV programs Mother helps you when you have problems with your studies. Your mother participates in parent activities organized by the school	Father studying middle school textbooks or tutorials on his own in order to tutor you Father buys books on educating children Your father attends lectures on education Father buys study books or materials for you Father assigns a study plan for you Father participates in parent activities organized by the school Father watches educational TV programs Father hires a tutor or finds someone to help you with your homework/Father does homework with you
Parental emotional engagement	Mothers encourage you when you do not do well on exams.	Your father encourages you when you do not do well on a test.
	Mother’s channeling of the difficult emotions you are experiencing in your studies Mother understands how you feel at school Your mother encourages you to do well in your exams. Your mother talks to you about how you approach your studies Your mother talks to you about things you are interested in at school.	Your father encourages you to study by using moral encouragement such as praise. Your father encourages you to do well in exams. Your father encourages you to study with material rewards Your father emphasizes the importance of learning through his own experiences or lessons. Your father tells you stories or philosophies about the importance of learning.
Parental management engagement	Mothers manage the time you spend watching TV Mother manages the time you spend playing with your friends Your mother manages the time you spend on the internet Mother asks you how you are doing with your homework Mother manages what you watch on TV Your mother manages your work schedule	Fathers manage the time you spend watching TV. Fathers manage the time you spend playing with your friends Father manages the time you spend on the internet Father asks you how your homework is done Father manages what you watch on TV Father manages your work and rest time

**Table 2:** Descriptive analysis.

Variable		Frequency	%
Student gender	Male	2618	52.6
	Female	2357	47.4
Grade	Grade 7	2729	53.7
	Grade 9	2349	46.3
Only child	Yes	1789	35.2
	No	3289	64.8
Left-behind type	Absent mother	710	14.0
	Absent father	2228	43.9
	Both parents absent	2140	42.1

**Table 3:** Comparison of left-behind child types.

Variable	Left-Behind Children				F	*p*
	Absent Mother (n = 710)	Absent Father (n = 2228)	Both Parents Absent (n = 2140)	Non-Left-Behind Children (n = 9769)		
Parental educational expectation	6.33 ± 1.72	6.48 ± 1.67	6.42 ± 1.61	6.66 ± 1.58	23.58 **	<0.01
Academic standard achievement	68.05 ± 9.09	69.62 ± 8.64	69.93 ± 8.47	70.33 ± 8.55	17.91 **	<0.01
Parental intellectual involvement	2.06 ± 0.99	2.13 ± 0.96	1.87 ± 0.99	2.29 ± 1.03	104.79 **	<0.01
Parental management involvement	2.27 ± 0.42	2.34 ± 0.38	2.33 ± 0.39	2.35 ± 0.39	13.48 **	<0.01
Parental emotional involvement	1.93 ± 0.53	1.96 ± 0.50	1.93 ± 0.53	2.03 ± 0.51	33.66 **	<0.01
Family economic condition	2.85 ± 0.62	2.89 ± 0.58	2.84 ± 0.59	3.04 ± 0.54	103.44	<0.01
Mother’s level of education	3.53 ± 1.87	3.69 ± 1.94	3.14 ± 1.62	3.99 ± 2.05	115.16	<0.01
Father’s level of education	3.83 ± 1.89	4.10 ± 1.93	3.49 ± 1.57	4.35 ± 2.06	118.62	<0.01
Relationship with mother	2.47 ± 0.68	2.71 ± 0.50	2.61 ± 0.58	2.74 ± 0.47	91.93	<0.01
Relationship with father	2.50 ± 0.61	2.44 ± 0.66	2.53 ± 0.60	2.61 ± 0.55	64.93	<0.01
Self-education expectation	3.91 ± 2.65	3.92 ± 2.48	3.89 ± 2.46	3.83 ± 2.52	1.04	0.38

Note: ** *p* < 0.01.

**Table 4:** The correlation matrix among variables.

	1	2	3	4	5
1. Academic standard achievement	1				
2. Parental intellectual involvement	−0.73 ***	1			
3. Parental management involvement	0.02	0.34 ***	1		
4. Parental emotional involvement	0.06 ***	0.32 ***	0.35 ***	1	
5. Parental educational expectation	0.35 ***	0.07 ***	0.15 ***	0.15 ***	1

Note: *** *p* < 0.001.

**Table 5:** Regression analysis of variable relationships.

Regression Equation		Overall Fitting Coefficient	Significance of Regression Coefficient	
Result Variable	Predictor	R2	β	t
Academic standard achievement	Parental educational expectation	0.18	0.35	25.87 ***
	Parental intellectual involvement		−0.11	−0.72 ***
	Parental management involvement		−0.01	−0.72
	Parental emotional involvement		0.05	3.18 ***
	Types of left-behind children		0.04	2.72 **
	Gender		0.19	14.5 ***
	Grade		0.04	2.77 **
	Only child		0.04	2.83 ***
Parental intellectual involvement	Parental educational expectation	0.06	0.04	4.98 ***
Parental management involvement	Parental educational expectation	0.03	0.03	10.16 ***
Parental emotional involvement	Parental educational expectation	0.04	0.05	10.74 ***

Note: ** *p* < 0.01; *** *p* < 0.001.

**Table 6:** Analysis of specific indirect effects.

Intermediate Path	Effect Size	Boot Standard Error	95% Confidence Interval	
			Lower Limit	Upper Limit
Total effect	1.80	0.07	1.67	1.94
Direct effect	1.82	0.07	1.68	1.95
Total indirect effect	−0.01	−0.01	−0.04	−0.02
Indirect effect 1	−0.04	−0.04	−0.06	−0.02
Indirect effect 2	−0.01	−0.01	−0.03	0.01
Indirect effect 3	0.04	0.04	0.01	0.06
C1	0.03	0.02	0.001	0.06
C2	−0.05	0.02	−0.08	−0.01
C3	−0.08	0.02	−0.11	−0.05

Notes: Boot standard error, Boot CI lower limit, and Boot CI upper limit refer to the standard error, lower limit, and upper limit of 95% confidence interval for indirect effects estimated by the deviance-corrected percentile Bootstrap method, respectively.

**Table 7:** Regression analysis of adjusted variable relationships.

**Regression Equation**		**Overall Fitting Coefficient**	**Significance of Regression Coefficient**	
**Result Variable**	**Predictor**	**R2**	**β**	**t**
Academic standard achievement	Parental educational expectation	0.18	0.34	25.50 ***
	Parental intellectual involvement		−0.12	−8.28 ***
	Parental management involvement		0.03	2.41 *
	Parental emotional involvement		0.04	2.62 **
	Types of left-behind children		0.03	2.48 *
	Gender		0.19	14.42 ***
	Grade		0.04	2.85 **
	Only child		0.04	2.73 ***
Parental intellectual involvement	Parental educational expectation	0.06	0.07	4.98 ***
Parental management involvement	Parental educational expectation	0.04	0.15	10.47 ***
Parental emotional involvement	Parental educational expectation	0.04	0.15	10.74 ***

Notes: * *p* < 0.05; ** *p* < 0.01; *** *p* < 0.001.

**Table 8:** Analysis of specific indirect effects after adjustment.

Intermediate Path	Effect Size	Boot Standard Error	95% Confidence Interval	
			Lower Limit	Upper Limit
Total effect	1.80	0.07	1.67	1.94
Direct effect	1.79	0.07	1.65	1.93
Total indirect effect	0.01	0.02	−0.02	0.04
Indirect effect 1	−0.04	0.01	−0.07	−0.02
Indirect effect 2	0.03	0.01	0.01	0.05
Indirect effect 3	0.03	0.01	0.01	0.05
C1	−0.01	0.003	−0.02	−0.01
C2	−0.01	0.003	−0.02	−0.01
C3	−0.0006	0.003	−0.01	−0.01

Note: Boot standard error, Boot CI lower limit, and Boot CI upper limit refer to the standard error, lower limit, and upper limit of 95% confidence interval for indirect effects estimated by the deviance-corrected percentile Bootstrap method, respectively.

## Data Availability

The data is available at http://ceps.ruc.edu.cn/xmjs/xmgk.htm, accessed on 12 March 2023.

## Appendix A. List of Survey Items

**Variables**		**Question of Investigation**
parents’ educational expectations		What are your parents’ educational expectations for you?
Types of left-behind children		Are there any immediate family members who are not currently living with you at home?
Parental education involvement	Parental intellectual involvement	Last week, did your parents urge you to study? “Check your homework”, “guide your homework”.
	Parental management involvement	Are your parents strict with you about the following things? Homework, tests, performance in school, going to school every day, what time you come home every day, who you are friends with, how you dress, how much time you spend online, how much time you watch TV.
	Parental emotional involvement	“How often do your parents discuss the following with you”—what’s going on at school, your relationship with your friends, your relationship with your teachers, your mind, your mind or troubles.
Academic achievement		Students’ midterm scores for the fall 2013 semester
